# Crystal Structures of a Plant Trypsin Inhibitor from *Enterolobium contortisiliquum* (EcTI) and of Its Complex with Bovine Trypsin

**DOI:** 10.1371/journal.pone.0062252

**Published:** 2013-04-23

**Authors:** Dongwen Zhou, Yara A. Lobo, Isabel F. C. Batista, Rafael Marques-Porto, Alla Gustchina, Maria L. V. Oliva, Alexander Wlodawer

**Affiliations:** 1 Macromolecular Crystallography Laboratory, Center for Cancer Research, National Cancer Institute, Frederick, Maryland, United States of America; 2 Departamento de Bioquímica, Universidade Federal de São Paulo, São Paulo, Brazil; 3 Unidade de Sequenciamento de Proteínas e Peptídeos - Laboratório de Bioquímica e Biofísica, Instituto Butantan, São Paulo, Brazil; University of Queensland, Australia

## Abstract

A serine protease inhibitor from *Enterolobium contortisiliquum* (EcTI) belongs to the Kunitz family of plant inhibitors, common in plant seeds. It was shown that EcTI inhibits the invasion of gastric cancer cells through alterations in integrin-dependent cell signaling pathway. We determined high-resolution crystal structures of free EcTI (at 1.75 Å) and complexed with bovine trypsin (at 2 Å). High quality of the resulting electron density maps and the redundancy of structural information indicated that the sequence of the crystallized isoform contained 176 residues and differed from the one published previously. The structure of the complex confirmed the standard inhibitory mechanism in which the reactive loop of the inhibitor is docked into trypsin active site with the side chains of Arg64 and Ile65 occupying the S1 and S1′ pockets, respectively. The overall conformation of the reactive loop undergoes only minor adjustments upon binding to trypsin. Larger deviations are seen in the vicinity of Arg64, driven by the needs to satisfy specificity requirements. A comparison of the EcTI-trypsin complex with the complexes of related Kunitz inhibitors has shown that rigid body rotation of the inhibitors by as much as 15° is required for accurate juxtaposition of the reactive loop with the active site while preserving its conformation. Modeling of the putative complexes of EcTI with several serine proteases and a comparison with equivalent models for other Kunitz inhibitors elucidated the structural basis for the fine differences in their specificity, providing tools that might allow modification of their potency towards the individual enzymes.

## Introduction

Members of the superfamily characterized by the β-trefoil fold [1] are structurally similar although their biological functions may be widely different [2]. Such functions may include chlorophyll binding [3], taste modification (miraculin [4]), binding to cytokine receptors (IL-1β [5]), tight binding to ribosomes (ricin [6]) or carbohydrate binding, exemplified by the *Clitocybe nebularis* lectin, CNL [7]. Prominent among them are plant protease inhibitors of the Kunitz type, the first one isolated by Kunitz [8] from soybeans and named soybean trypsin inhibitor (STI). A complex of STI with trypsin provided the first example of the interactions between a member of this family and its target enzyme [9], [10]. STI and related proteins mainly inhibit serine proteases, although some also inhibit cysteine and aspartic proteases [11]. Plant Kunitz inhibitors are characterized by molecular mass of about 20,000 Da (for the whole protein or a domain), low content of cysteine residues, and the presence of one or two reactive sites that are responsible for their inhibitory activity. They are frequently present in several polymorphic variants, exemplified by the STI isoforms, Tia and Tib, which differ by nine amino acids [12].

A Kunitz-type inhibitor that was first isolated from *Enterolobium contortisiliquum* was named EcTI [13]. It was shown that EcTI inhibits trypsin, chymotrypsin, plasma kallikrein, plasmin, human neutrophil elastase, and Factor XIIa in the stoichiometric ratio 1∶1, but not thrombin, bovine pancreatic elastase, or Factor Xa [13], [14]. The primary structure of EcTI was determined by automated Edman sequencing [15] after digestion of the protein with trypsin, chymotrypsin, and protease from *S. aureus*. A molecule of EcTI was shown to contain two polypeptide chains consisting of 134 and 40 amino acid residues each, covalently bound through a disulfide bond, with one residue (116) unidentified [13]. The protein was subsequently crystallized and a 2 Å data set was collected [16], but full structure determination was not completed.

An investigation of the biological properties of EcTI has concluded that this inhibitor showed no effect on the proliferation of gastric cancer cells or fibroblasts but inhibited the adhesion, migration, and cell invasion of gastric cancer cells, with no effect on the adhesion of fibroblasts. EcTI was shown to decrease the expression and disrupt the cellular organization of molecules involved in the formation and maturation of invadopodia. Gastric cancer cells treated with EcTI exhibited a significant decrease in intracellular phosphorylated Src and focal adhesion kinase, integrin-dependent cell signaling components. It was speculated that the mode of such activity of EcTI involves inhibition of the invasion of gastric cancer cells through alterations in integrin-dependent cell signaling pathways [14], [17].

In this work, we investigated the structural basis of the activity of EcTI by determining its crystal structure in the unbound and trypsin-bound form. Modeling of the mode of binding of EcTI to other serine proteases was used in order to elucidate the ability or lack thereof to inhibit their activity.

## Results and Discussion

### Crystal Structures of EcTI

**Figure 1:**
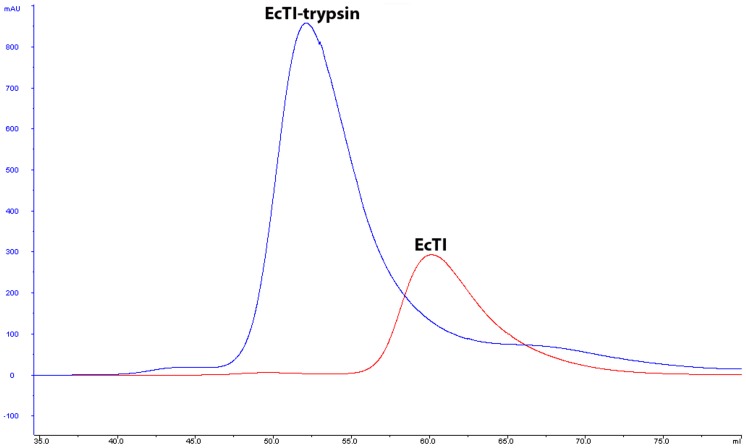
Preparation of the EcTI-trypsin complex. Free EcTI and its complex with trypsin were run on the same Sephacryl S-100 HR column, with the same buffer. The two curves were overlaid, with the blue one representing EcTI-trypsin and the red one representing free EcTI.

The plant protease inhibitor EcTI from *Enterobobium contortisiliquum* is a classical trypsin inhibitor belonging to the Kunitz family. It exhibits potent inhibition of bovine pancreatic trypsin and a number of other serine proteases (**Table 1**), but does not inhibit several related enzymes, such as Factor Xa. In this study we evaluated the ternary structure of the inhibitor and its interactions with serine protease trypsin. Formation of the EcTI-trypsin complex was tested on an analytical scale using a small column BioSep-SEC-S 3000 (Phenomenex), indicating strong binding of EcTI to bovine trypsin. A larger amount of the EcTI-trypsin complex was prepared with a Sephacryl S-100 HR column. A reasonably large single peak corresponding to EcTI-trypsin complex was observed (**Fig. 1**). The complex was subsequently crystallized and the crystal structure was determined at 2.0 Å resolution. The final model contains a single complex in the asymmetric unit. The electron density for the trypsin molecule is complete and all 223 amino acid residues are traced in the final model (for consistency with the previous studies we used standard chymotrypsin numbering, 16–245). In the EcTI molecule residues belonging to the loop region 111–113, the C terminus of the longer polypeptide chain (135–136), the N terminus of the shorter chain (137–140), and the C terminus of the shorter chain (175–176) were not included in the final model due to the lack of electron density.

**Table 1:** Inhibitory properties of EcTI and the related inhibitors.

	EcTI	STI	TKI*
Bovine trypsin Ki (nM)	0.88 (±0.25)	1.29 (±0.73)	3.2
Bovine chymotrypsinKi (nM)	1.11 (±0.67)	70.29 (±31.67)	
Human plasmakallikrein Ki (nM)	6.15 (±0.98)	5.70 (±3.54)	
Human plasminKi (nM)	9.36 (±0.92)	192.10 (±78.37)	
Human factor XaKi (nM)	n.i.	1367.59 (±218.20)	220
Human factor XIIaKi (nM)	81.81 (±14.89)	n.i.	
Human plasminogenKi (nM)	n.i.	n.i.	
Porcine pancreatickallikrein	n.i.		
Bovine pancreaticelastase	n.i.		
Human neutrophilelastase Ki (nM)	55		

n.i – no inhibition. The values in parentheses correspond to the standard deviations of the calculated data. Blank fields – data not collected or available.

* Taken from Patil et al. [19].

The structure of free EcTI was refined to a higher resolution of 1.75 Å. The final model contains two inhibitor molecules, two imidazole molecules, two glycerol molecules, and 244 water molecules. Molecule A consists of residues 1–134 and 141–174, whereas molecule B contains residues 1–134 and 141–173. A loop region containing residues 23–26 of monomer B is in weak electron density, but is still included in the final model, whereas the density for the loop containing residues 111–113, not traced in the complex, is very clear.

Both structures were validated with the program *MolProbity* [18] which showed over 95% residues falling within the favored area and no residues in the disallowed regions, indicating acceptable quality of the structures.

### Redetermination of the Sequence of EcTI

**Figure 2:**
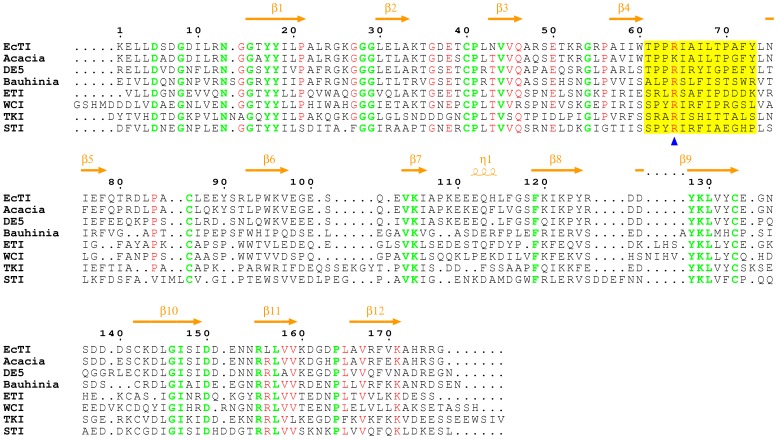
Sequence alignment of EcTI and related serine protease inhibitors, partially structure-based (the structures of Acacia, DE5, and Bauhinia (rows 2–4) have not been determined). The top two sets of rows correspond to the long chain in EcTI, and the third set of rows represents the short chain. The secondary structure elements of EcTI are shown on top of the alignment in orange. Residues identical in all inhibitors are colored green and those identical in some, red. The reactive loop regions are highlighted in yellow, and a blue triangle indicates the position of P1 residue.

Strikingly, the high resolution structure of free EcTI indicated that the crystallized protein is a new isoform, since its sequence differs in a number of places from the one reported previously (Accession ID P86451 [13]). The measured molecular mass of the disulfide-linked EcTI molecule, obtained with an electrospray mass spectrometer, was 19851.5 Da, whereas the mass calculated from the previously published sequence was only 19475.2 Da. After reduction of the disulfide bonds the molecular mass of the C-terminal chain was 4440.2 Da, lower than the isotopically averaged calculated 4482.9 Da. The latter discrepancy was resolved by replacing Arg154 with a leucine, very clearly delineated in the electron density map in both molecules. This replacement changed the calculated mass of this chain to 4439.9, in excellent agreement with the experimentally determined value. A larger number of changes were required to determine the sequence of the N-terminal chain. Two residues (Gln102 and Glu111) had to be inserted, and the identity of 17 residues changed. The isotopically averaged mass of the fully reduced N-terminal chain of EcTI is now calculated as 15416.5, compared to the experimentally determined 15418.5. The sequence of this isoform of EcTI and its comparison with the sequences of other Kunitz inhibitors is shown in **Fig. 2**.

### Description of the Three-dimensional Structure of EcTI

**Figure 3:**
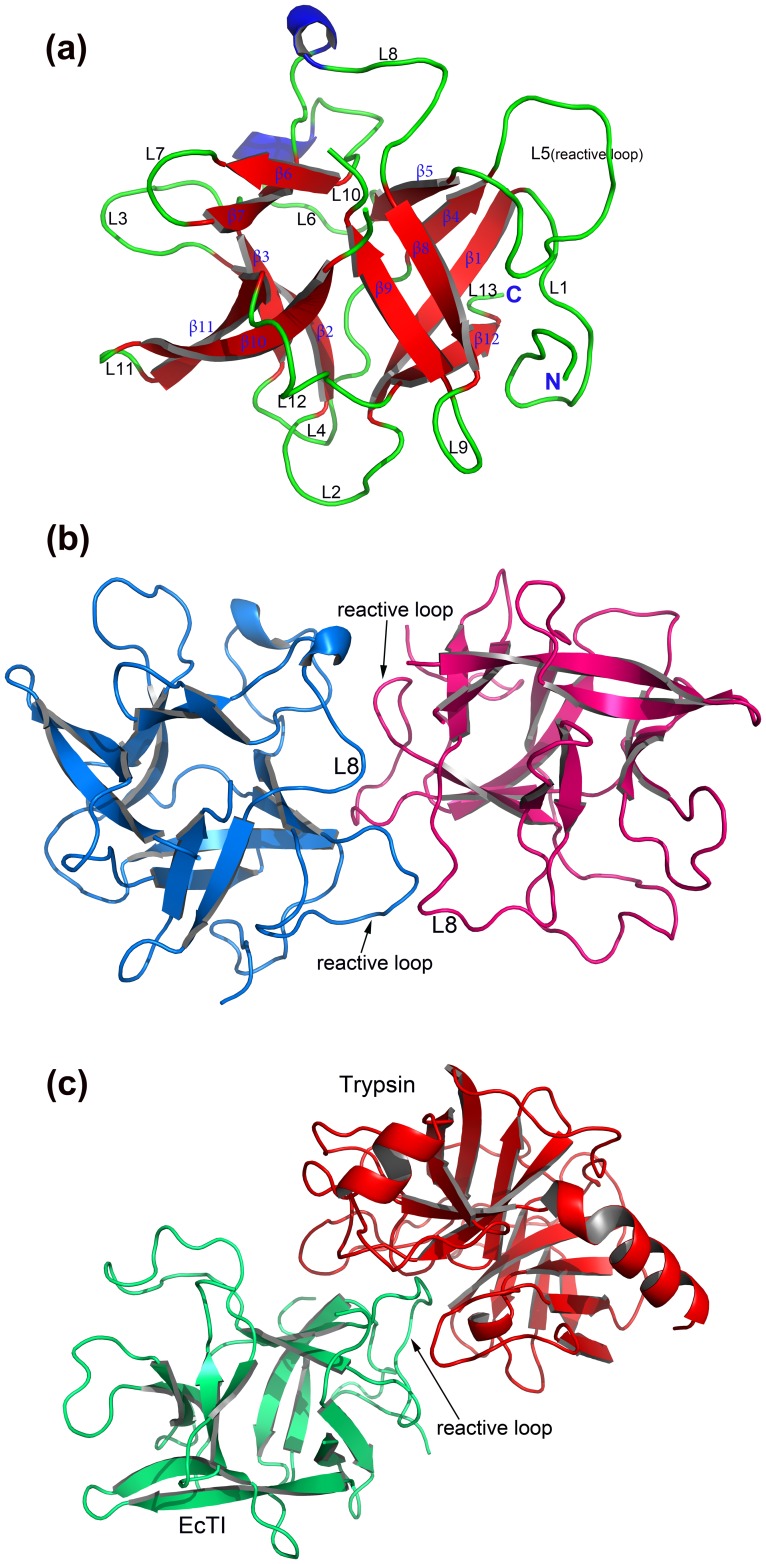
EcTI and its complex with trypsin. (a) Ribbon representation of the overall three-dimensional structure of EcTI. β-strands labeled β1–12 are shown in red, and loops labeled L1–13 are in green. Loop L10 is only marked for consistency with the other related structures, since the main chain is broken in this region. (b) Pseudo-dimer of the two crystallographically independent molecules of EcTI in the asymmetric unit. The main contact loops are labeled. (c) Overall structure of the EcTI-trypsin complex shown as a cartoon diagram. EcTI is colored green, while trypsin is red, and the reactive loop is labeled.

The fold of EcTI is a β-trefoil, typical for the STI-related family of Kunitz inhibitors. Such a fold contains twelve β-strands (labeled β1 to β12) arranged into three structurally similar units related by pseudo-threefold symmetry (**Fig. 3a**). A β-barrel which creates a significant hydrophobic core is formed by all twelve β-strands. Loop L10 is present only nominally, since this part of the structure includes the C terminus of the longer chain and the N terminus of the shorter chain, both partially disordered. The reactive loop (L5) responsible for the inhibitory properties is located between β-strands 4 and 5. Since the crystals of free EcTI contain two molecules in the asymmetric unit, it is possible to evaluate the influence of crystal packing on its structure. The root-mean-square deviation (RMSD) between the coordinates of the two molecules is 0.36 Å, calculated for 150 pairs of superimposed Cα atoms. A significant difference was observed at the loop L8 between β-strands 7 and 8, most likely caused by the interactions between two molecules, since the L8 loops of both of them are involved in forming dimer contacts. Other contact areas include loops L5 (reactive loop), L10, and L11 of molecule A, and loop L1, strand β1, reactive loop L5, and the loop L6 of molecule B (**Fig. 3b**). However, since the protein migrates on gels as a monomer (**Fig. 1**), the observed dimer is a crystallization artifact.

Only a single EcTI-trypsin complex is present in the asymmetric unit of its crystals. EcTI binds to the active site of trypsin through its reactive loop (**Fig. 3c**). The molecules of EcTI in the complex are very similar to their counterparts in the unbound EcTI structure. The RMSDs between the complexed EcTI and molecules A and B of the free inhibitor are 0.305 and 0.245 Å, respectively. The most significant difference between the free and complexed EcTI is the conformation of the loop L8 (residues 107–117). Residues 110–113 are disordered in the complex while well ordered in both molecules of the free inhibitor. However, in the latter two structures the conformation of loop L8 is significantly different. In molecule B the conformation of this loop is closer to the one observed in the complex structure, whereas in molecule A its conformation is very different, most likely due to crystal contacts. In the structure of the complex residues 114–116 of the inhibitor are significantly shifted (as much as 4.3 Å for Cα of Leu115), compared to their position in the structure of free inhibitor in molecule B. This shift is most likely driven by multiple interactions with residues 97–99 of trypsin and by hydrophobic interactions with the side chain of Trp215.

**Figure 4:**
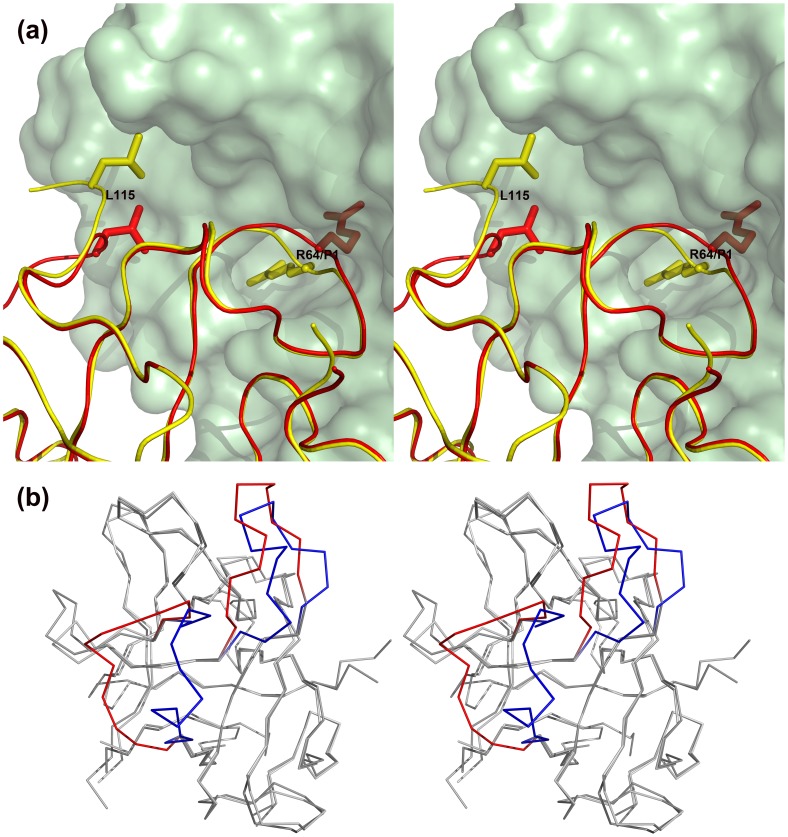
Superposition of EcTI in free and bound state. (a) Interface between EcTI (yellow ribbon) and trypsin (green space filling representation) in the inhibitor-trypsin complex. The side chains of Arg64 and Leu115 of EcTI are shown as sticks. A superimposed tracing of molecule B of free EcTI is shown in red, clearly indicating that the conformational changes in the reactive loop around Arg64 are needed in order to avoid clashes and allow the proper fit of its side chain into the S1 pocket of the enzyme. (b) Steroview of the superposition of trypsin-bound EcTI and STI (PDB ID: 1AVW). Color highlights are for the loops which are most different between these two proteins, blue for EcTI and red for STI, whereas other parts of the chain are gray.

Similarly to the situation observed in the STI-porcine trypsin complex [10], formation of the EcTI-trypsin complex does not lead to significant overall conformational changes of the reactive loop. Structural differences are primarily limited to the main chain in the vicinity of the P1 residue Arg64 which occupies the S1 pocket of trypsin. As a result, the orientation of the side chain of Arg64 has changed upon binding allowing a perfect fit of this residue in the S1 pocket (**Fig. 4a**). However, we have not observed the lowering of the B-factors of the reactive loop residues upon enzyme binding, reported for other similar complexes [10], [19]. A comparison of the trypsin component of the complex with the structure of the inactivated enzyme containing monoisopropylphoshoserine instead of the catalytic Ser195 (PDB ID: 5PTP) indicates the absence of any significant conformational changes in the enzyme that could be traced to binding of EcTI (RMSD 0.50 Å for a superposition of all 223 Cα atoms).

A molecule of EcTI contains two disulfide bonds, one intra-chain and one connecting two chains. The intra-chain bond involves Cys40 and Cys86, connecting loops L3 and L6, and an equivalent disulfide bond is observed in most Kunitz-type inhibitors [20]. The second disulfide bond is formed by Cys133 and Cys142, covalently connecting the two polypeptide chains, thus providing additional stability to the β-trefoil. That bond is also conserved in most other Kunitz inhibitors, although it is usually intra-chain rather than inter-chain. However, since the crystal structures of EcTI were obtained under mildly reducing buffer conditions, the disulfide bond Cys40-Cys86 was broken in molecule A of the free EcTI, as well as in the inhibitor molecule in the EcTI-trypsin complex. This observation indicates that the disulfide bond Cys133-Cys142 plays a more important role in stabilizing the β-trefoil fold, whereas the disulfide bond Cys40-Cys86 may not be as necessary for its stability.

### Structural Comparison of EcTI with Other Inhibitors

A sequence alignment of EcTI and its selected homologues is shown in **Fig. 2**. The alignment utilized three-dimensional structures of the proteins if known, otherwise only their primary structures. EcTI shows the highest sequence identity of 87% with a trypsin inhibitor from *Acacia confusa*. The sequence identity between EcTI and DE5, a trypsin inhibitor from *Adenanthera pavonina*, is 67%, and the identities between EcTI and *Bauhinia variegata* trypsin inhibitor, ETI, WCI, TKI, and STI are 45%, 38%, 34%, 44%, and 40%, respectively. However, crystal structures are not available for the most closely related evolutionary counterparts of EcTI, trypsin inhibitors from *Acacia confusa* and *Adenanthera pavonina*. Not surprisingly, the sequence alignment shows that the most highly conserved residues are located in the β-strand regions, being responsible for creation of the common β-trefoil fold. On the other hand, the presence of residues that are not conserved allows these STI family members to exhibit different biological functions.

Other Kunitz-type inhibitors with experimentally determined structures that share the β-trefoil fold with EcTI are STI, TKI, ETI, and WCI. When the structure of EcTI was superimposed with STI and TKI, the β-trefoil portions were overlaid fairly well, with the respective RMSD values of 0.44 Å for the 101 pairs of superimposed Cα atoms of STI and 0.49 Å for 99 atom pairs of TKI. However, significant differences are found at the loop regions. For example, the tracing of loops L6 and L8 is completely different in EcTI and STI, with the largest distance between the L6 loops being as much as 14.6 Å (**Fig. 4b**). However, the differences in the reactive loops of these two proteins are relatively minor, with the loops L5 retaining very similar shape and the largest deviation not exceeding 1.5 Å (see below).

### The Reactive Site

**Figure 5:**
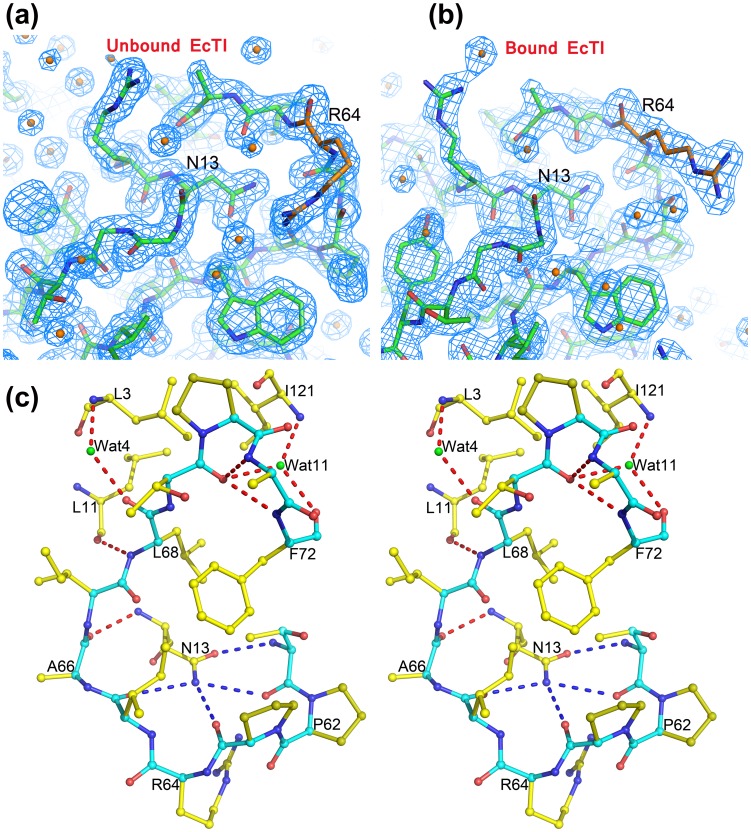
The reactive loop of EcTI. (a,b) 2Fo-Fc electron density maps contoured at 1.2σ of the reactive loop area of (a) free EcTI and (b) its complex with trypsin. (c) A steroview of the hydrogen networks of the reactive loop region of a molecule of free EcTI. Blue dashed lines indicate the hydrogen bond network formed by the side chain of Asn13 and the interacting residues, and red dashed lines indicate the hydrogen bond network formed by the reactive loop region residues and their partners. Green spheres represent water molecules.

As found in most known Kunitz-type inhibitors [2], EcTI has a single reactive site located on loop L5, between β-strands 4 and 5. An exception is the recently published complex structure of a double-headed serine arrowhead protease inhibitor, API-A complexed with two molecules of trypsin, in which two reactive sites are located in loops L6 and L10 [21]. However, the latter protease inhibitor may represent a different evolutionary pathway. The electron density maps for the reactive loops of both the free EcTI (**Fig. 5a**) and in a complex with trypsin (**Fig. 5b**) are well defined, indicating the presence of stable conformations. We did not observe lowering of the B-factors of the backbone of the reactive loop upon complex formation, unlike what was reported for STI and TKI [10], [19]. This difference is very likely due to the involvement of the reactive loops of both EcTI molecules located in an asymmetric unit of the crystals of free inhibitor in dimer formation. However, the B-factor of the side chain of Arg64, the P1 residue of the inhibitor, is significantly lower in the complex than in the free inhibitors, due to numerous interactions of its extended side chain with the residues comprising the tight S1 pocket in the active site of trypsin. These interactions include a strong ion pair with Asp189, located at the bottom of the pocket. The side chains of Arg64 are exposed to the solvent in both EcTI molecules present in an asymmetric unit of the crystals of the free inhibitor.

As discussed above, despite the extensive structural conservation of the overall β-trefoil folds of Kunitz-type inhibitors and excellent superposition of their β strands, conformations of some loops are quite different. However, conformations of the reactive loops L5 are very similar, although their amino acid sequences are not highly conserved. A network of hydrogen bonds formed by the side chain of Asn13 (STI numbering) with the reactive loop residues was thought to play an important role in stabilizing the conformation of the reactive loop [10]. The equivalent residue in EcTI is also Asn13, which similarly forms a network consisting of several hydrogen bonds. In addition, seven hydrogen bond interactions (Ala66-O-Asn13-N, Leu68-N-Leu11-O, Leu68-O-H_2_O-Leu3-N, Thr69-O-Ala71-N, Thr69-O-Phe72-N, Thr69-O-H_2_O-Ile121-N, and Phe72-O-H_2_O-Ile121-N) might play an essential role in stabilizing the conformation of the reactive loop (**Fig. 5c** **)**. These hydrogen bonds involve residues extending from P2′ to P8′ and their immediate environment.

Superposition of the structures of the complexed and free EcTI reveals that there is no significant conformational change of the reactive loop upon binding to trypsin. Taking monomer B of free EcTI as a reference, only the Cα atoms of the P1, P2, and P1′ residues exhibit minor shifts, whereas the remaining residues of the reactive loop are in almost exactly the same conformation. Among these three residues, the largest movement (1.5 Å) involves the P1 residue (**Fig. 4a**). The conformational changes can be further elucidated by the changes in the dihedral angles. The P1, P2, and P1′ residues of free EcTI monomer B have dihedral angels (φ/ψ) of −80.6/75.4, −60.2/156.1, and −133.6/140.0, respectively, whereas the equivalent residues in the complex have dihedral angles of −93.0/40.9, −64.7/142.6, and −76.8/156.0, respectively. The largest changes are for the dihedral angle ψ of the P1 residue and φ of the residue at P1′. However, this minor conformational change is indispensable for complexation. **Fig. 4a** shows the potential clashes between the reactive loop of free EcTI and trypsin.

**Figure 6:**
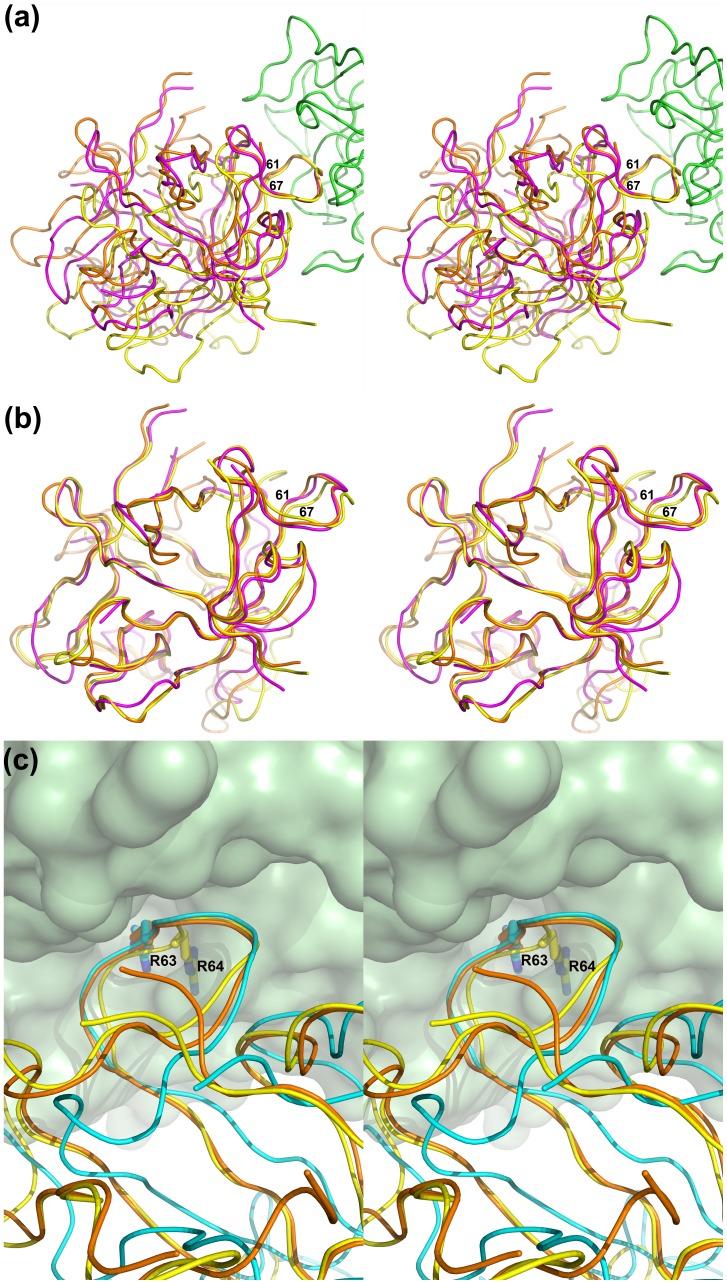
Rigid body movement of the Kunitz inhibitors bound to trypsin. (a) Superposition of the trypsin complexes with EcTI, STI, and TKI based on the Cα coordinates of trypsin reveals overall rigid body shifts of the inhibitors. The tracing of the trypsin chain in the complex with EcTI is shown as green ribbon, EcTI is yellow, STI red, and TKI magenta. (b) Superposition of EcTI, STI, and TKI based on their Cα coordinates (colored as in panel (a)) reveals a small change in the orientation of the reactive loop (residues 61–67). (c) Superposition of EcTI bound to trypsin with the STI complex. STI is shown in orange and trypsin in space-filling green. In order to show the reason for the rigid body movement of EcTI it is shown twice, in yellow when superimposed directly on STI, and in blue when the trypsin molecules of the two complexes were superimposed.

Whereas the reactive loops of EcTI, STI, and TKI bound to trypsin adopt almost identical conformations when the trypsin components of the complexes are superimposed, the inhibitors themselves are not well superimposed despite their high structural similarity (**Fig. 6a**). If the inhibitor components of the complex are superimposed instead, we observe very accurate overall superposition of their β sheets, whereas the reactive loops are not well superimposed (**Fig. 6b**). Thus in a superposition in which both components, trypsin and the inhibitors, were to be individually and optimally superimposed using the STI complex as a guide, the reactive site of EcTI would be misaligned in relation to the active site of trypsin. In particular, the side chain of Arg64 would clash with the wall of the active site of trypsin, instead of occupying the proper S1 binding pocket. However, when the actual orientation of EcTI bound to trypsin is considered, the superposition of the reactive loops as well as of the side chains of Arg64 is excellent (**Fig. 6c**). Therefore, the only way to avoid such clashes while preserving conserved conformation of the reactive loop (as observed in various Kunitz inhibitor complexes with trypsin) is through a rigid body movement of the inhibitor molecule **(** **Fig. 6a,c**). The inhibitor molecules are rotated by ∼10° relative to each other in the EcTI and STI complexes with trypsin, a value similar to the rotation of eglin-C in its complexes with subtilisin and thermitase [**22**].

### Interface between the Enzyme and the Inhibitor

**Figure 7:**
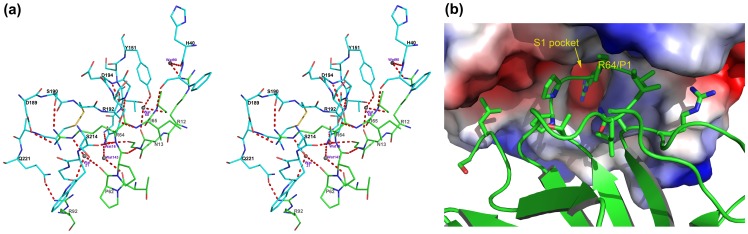
Close-up of the interactions of EcTI and trypsin. (a) Steroview of the region of interactions, with residues belonging to trypsin shown as cyan sticks and the disulfide bond colored yellow. The residues of EcTI are shown as green sticks and the gray spheres represent water molecules. Hydrogen bonds are indicated by red dashed lines. (b) Interface of the EcTI-trypsin complex, with EcTI shown as a green ribbon with selected side chains in stick representation, whereas the surface of trypsin is colored according to charge (blue positive, red negative, gray uncharged).

EcTI belongs to the family of substrate-like inhibitors characterized by the presence of a reactive site loop in canonical conformation [10]. Its reactive site adopts a classical noncovalent “lock and key” inhibitory mechanism. The side chain of the P1 residue Arg64 acts exactly like a key inserting into the active site of trypsin. The whole contact area is 987 Å^2^ as calculated with the program PISA [23]. The interaction pattern between EcTI and trypsin is shown in **Fig. 7a** and is summarized in **Table 2**. Eight EcTI residues make hydrogen bonds to trypsin, and eleven residues in EcTI are involved in hydrophobic interactions with trypsin. Not surprisingly, a majority of these residues are located in the reactive loop. Additional interactions are provided by the surrounding residues from the N terminus, as well as loops L6 and L8. The key P1 residue, Arg64, interacts with seven residues of trypsin. The NH2 atom of the guanidinium group of Arg64 makes an ionic interaction with the carboxylate group OD2 of trypsin Asp189 at a distance of 2.99 Å, and also makes a hydrogen bond with the carbonyl O atom of trypsin Gly219. The carbonyl O atom of Arg64 accepts two hydrogen bonds from the main chain amide nitrogens of Gly193 and Ser195 in trypsin, whereas the main-chain amide of Arg64 is engaged in forming hydrogen bonds with the carbonyl oxygens of trypsin Ser195 and Ser214. The NH1 group interacts with the hydroxyl group OG of trypsin Ser190. In addition, the nitrogen NE atom makes indirect hydrogen bonds with the carbonyl oxygens of three trypsin residues, Gly216, Gly219 and EcTI Pro62, mediated by a water molecule. In total, Arg64 forms a salt bridge with Asp189 of trypsin, as well as six direct hydrogen bonds and three indirect hydrogen bonds. In the reactive loop region, other contributing residues include Thr61 (P4), Pro62 (P3) and Ala66 (P2′), which form hydrogen bonds with several residues in trypsin. At the N-terminus of EcTI, residues Arg12, Asn13, and Gly14 are involved in water-mediated interactions with residues in trypsin. The NH1 atom of Arg92, a residue residing in the L6 loop, forms a hydrogen bond with the carbonyl OE1 of Gln221 in trypsin. Apart from these polar residues, eleven residues in EcTI contribute to hydrophobic interactions with trypsin. They are Ile10, Gly15, and Thr16 from the N terminus, Ile58, Trp60, Pro63, Ile65 and Ile67 from the reactive loop, and Ser114, Leu115, and Phe116 from loop L8. For the trypsin component, contact area mainly comprises the S1 pocket, occupied by the side chain of P1 residue Arg64 (**Fig. 7b**), and the surrounding area. The presence of two consecutive proline residues in the reactive loop of EcTI makes the sequence of this loop unique among other Kunitz inhibitors, but does not change the canonical binding mode.

**Table 2:** Interactions between EcTI and trypsin.

EcTI	Trypsin	Water	Distance(Å)
Arg12-NH1		Wat90	2.51
	His40-O	Wat90	2.85
Asn13-O		Wat68	2.74
	Tyr151-OH	Wat68	2.46
Gly14-O		Wat16	2.71
	Gly148-N	Wat16	3.10
Thr61-O		Wat143	2.71
	Gln192-NE2	Wat143	3.0
Pro62-O		Wat21	3.16
	Gly219-O	Wat21	2.99
	Gly216-O	Wat21	3.05
Arg64-O	Gly193-N		2.82
O	Ser195-N		2.96
N	Ser214-O		3.11
N	Ser195-OG		2.86
NH1	Ser190-OG		2.85
NH2	Asp189-OD2		2.99
NH2	Gly219-O		2.64
NE		Wat21	2.69
	Gly219-O	Wat21	2.99
	Gly216-O	Wat21	3.05
Ala66-O		Wat68	2.95
	Tyr151-OH	Wat68	2.46
Ala66-N	Phe41-O		3.02
Arg92-NH1	Gln221-OE1		2.43

### Structural Basis of Inhibitory Activities

Crystal structure of the EcTI-trypsin complex reveals the binding mode of this inhibitor to trypsin, demonstrating the conserved conformation of the reactive loop of the inhibitor docked in the active site of the enzyme. The side chain of the P1 residue, Arg64, is involved in charge-charge interactions with Asp189 in the S1 pocket of trypsin. That highly conserved interaction defines the primary specificity for the recognition of the trypsin-like serine proteases by Kunitz type inhibitors. A comparative analysis of the interactions reported here with those observed in the complexes of the other enzymes with similar inhibitors may provide a structural insight into the specificity and the potency of the inhibitors towards the individual enzymes.

**Figure 8:**
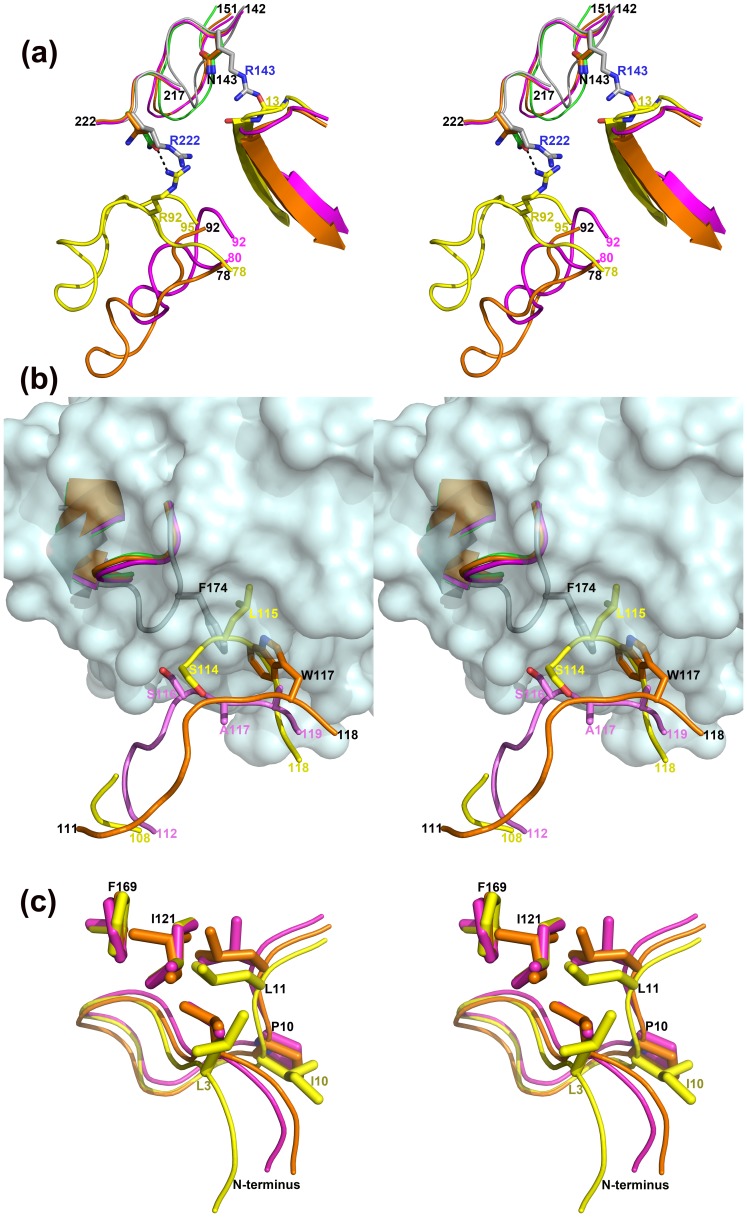
Structural basis for the differences in the inhibitory properties of Kunitz inhibitors towards different serine proteases. (a) Two possible areas of conflict between EcTI and Factor Xa. Factor Xa (gray) is superimposed on trypsin (green) complexed with EcTI (yellow). Complexes of STI and TKI are superimposed on the basis of their trypsin components (orange and magenta, respectively). (b) The third possible area of conflict between EcTI and Factor Xa. A modeled surface of Factor Xa (PDB ID: 2JKH) is gray. The colors for other molecules are as in (a). (c) Different orientation of the N terminus in EcTI (yellow) compared to STI (magenta) and TKI (orange).

EcTI is a potent inhibitor of several serine proteases including trypsin, chymotrypsin, plasma kallikrein, and plasmin, and a slightly less potent inhibitor of Factor XIIa (**Table 1**). However, EcTI does not inhibit Factor Xa (FXa) and human plasminogen (**Table 1**). Factor Xa is a serine protease involved in the coagulation cascade and is a potential target for the development of new antithrombotics. The structure of FXa is comprised of two chains, with the heavy chain exhibiting 37% sequence identity compared to bovine pancreatic trypsin. The RMSD between the heavy chain of Factor Xa in its atomic-resolution structure (PDB ID: 2JKH; [24]) and bovine trypsin (this work) is 0.65 Å for 169 Cα pairs, indicating very high conservation of their overall folds. The S1 pockets of both enzymes contain the negatively-charged Asp189, defining the primary specificity requirement of the presence of either arginine or lysine residue in the P1 position in the inhibitor. However, although Factor Xa is inhibited with low micromolar Ki by STI (**Table 1**) and with 220 nM Ki by TKI, a Kunitz type trypsin inhibitor from tamarind [19], it is not inhibited by EcTI. Superposition of the structure of FXa (PDB code 2JKH) on the trypsin molecule in the EcTI complex based on the Cα atoms reveals three potential areas of conflict for the EcTI binding to FXa. The first area includes the loop comprising residues 78–95 in EcTI, which adopts significantly different conformation than that in TKI and STI (**Fig. 8a**). As a result, a fragment of this loop that contains Arg92 would be placed very close to the loop 219–223 in FXa. The latter loop contains Arg222, and these two positively charged residues would collide. A trypsin residue that is structurally equivalent to Arg222 is Gln221, involved in favorable interactions with EcTI by forming a hydrogen bond with Arg92. Therefore, binding of EcTI to FXa would lead to potential clashes and the loss of a favorable interaction in this area when compared with binding of this inhibitor to trypsin. On the other hand, binding of TKI and STI to FXa would not lead to any negative effects in this area, due to different conformations of the loop 78–95.

The second site is located at the interface between the loops 13–15 in the inhibitors and 142–151 in the enzymes. In contrast to the previously described site, the conformation of this loop in different inhibitors is well preserved. This loop plays an important role maintaining the conformation of the reactive loop in the structures of all three inhibitors in the complexes with trypsin. Substitution of Asn143 in trypsin by Arg in FXa creates a potential clash with the main chain of the loop in the vicinity of Asn13. The latter residue forms several hydrogen bonds with the backbone of the reactive loop in the inhibitors. Any conformational changes in this area which would be needed to find a compromise solution for the inhibitor binding to FXa might affect the inhibition constant, and indeed, the binding of STI and TKI to FXa is weaker than to trypsin (**Table 1**).

The third site is the interface between the loops in the inhibitors and the enzymes with different conformations in both counterparts. Loop 171–175, following the conserved helical turn 167–171, has a different conformation in FXa then in trypsin. Loop 110–115 in EcTI and the corresponding fragments in STI and TKI differ in their conformation and sequence. Although the fragment 111–113 of this loop in EcTI is disordered, the conformation of the loop for the residues 114–115 is sufficiently similar between the bound and unbound state of the inhibitor to provide confidence for using this fragment for modeling purposes. Simple modeling shows clashes between the main chain of the latter and the side chain of Phe174 of FXa (**Fig. 8b**). Interestingly, the sequence of this fragment in TKI has only residues with small side chains (Ser116, Ala117, Ala118), and does not have any steric problems with accommodating a different conformation of the loop 172–175 in FXa. However, STI has large Trp side chain at the position 117, which may have potential clashes with the side chain of Phe174 in FXa (**Fig. 8b**). This observation is in a good agreement with the differences in the inhibition constants for STI and TKI (**Table 1**) and may explain why STI is worse inhibitor of FXa than TKI.

Significant differences in the inhibition constants for EcTI and STI are seen towards chymotrypsin and plasmin (**Table 1**). EcTI is between one and two order magnitude more potent towards these enzymes than STI. Possible steric problems that could be elucidated upon the simple modeling of STI and EcTI inhibitors in the active sites of these two enzymes might be addressed to the differences in the positions of their N termini. When the three inhibitors are superimposed, the orientation of the N termini in STI and TKI is similar, whereas the N terminus of EcTI is shifted (**Fig. 8c**). These differences can be related to the presence of the longer side chain of Leu3 and Ile10 in EcTI compared to shorter valine and proline side chains, occupying structurally equivalent positions in STI and TKI, respectively. These residues are involved in numerous hydrophobic interactions within the hydrophobic core comprising residues Leu11, Ile121 and Phe169. Incorporation of the longer side chains of Leu3 and Ile10 requires some adjustment in the orientation of the residues for optimization of the hydrophobic interactions within the core, resulting in the shift of the N termini in EcTI (**Fig. 8c**).

The differences in the orientations of the N termini of the inhibitors in the complexes are much more prominent due to the rigid body movement of the inhibitors described above. In a model of the complex of STI with chymotrypsin (PDB ID: 1YPH), Phe566 of STI would collide with Phe39 in chymotrypsin, and the changes in the orientation of its side chain are restricted by close proximity of the side chain of Phe502 on the N terminus of STI. The N terminus of EcTI is not close to the enzyme and instead of bulky Phe566, this inhibitor has an isoleucine at this position.

The loop 605–613 in plasmin (PDB ID: 1BML; [25]), equivalent to the loop 59–62 in trypsin, has a four-residue insertion. Two of them, Glu606 and Lys607, would be involved in clashes with the residues of STI in a modeled complex. In particular, the side chain of Lys607 collides with the positively charged N terminus of STI, whereas the N terminus of EcTI is located ∼8 Å away. In summary, these considerations could explain why EcTI is a better inhibitor of chymotrypsin and plasmin than STI (**Table 1**).

Whereas the modeling which was used in this study in an effort to explain the biochemical data was very simple, the resulting ideas of the structural basis for the differences in the potency of the inhibitors could be tested further. It should be possible to either increase the specificity of the individual inhibitors for a particular enzyme, or to make an inhibitor more promiscuous and able to efficiently inhibit a larger variety of targets.

## Materials and Methods

### Preparation and Purification of EcTI-trypsin Complex

Purification of the inhibitor EcTI by itself was carried out as previously described [13], [16]. For preparation of the complex, equimolar amounts of EcTI and bovine pancreatic trypsin (Sigma) were mixed and incubated overnight at room temperature in 20 mM Tris pH 8.0, 200 mM NaCl, 5% glycerol, and 6 mM β-mercaptoethanol. The mixture was then applied onto a Sephacryl S-100 HR (GE Healthcare) column pre-equilibrated with the same buffer. Fractions of 2 ml were collected at a flow rate of 0.2 ml/min. Samples pooled from different peaks were verified by SDS-PAGE. The fractions corresponding to EcTI-trypsin complex were selected and concentrated to around 10 mg/ml. The freshly prepared complex sample was aliquoted and immediately used for crystallization trials. The remaining aliquots were stored at −80°C for future optimization.

### Amino Acid Sequence of the Isoform of EcTI

Fitting of the published amino acid sequence of EcTI [13] to the electron density maps (see below) indicated disagreement between the identity of a number of amino acids and the shape of the corresponding density, necessitating reassessment of the primary structure of this protein. Molecular masses of the native and reduced EcTI were evaluated with the Agilent 1100 electrospray liquid chromatograph/mass spectrometer. The molecular mass of the reduced heavy (N-terminal) chain of EcTI was 15418.5 Da, and for the light (C-terminal) chain 4440.23 Da. Neither of these masses agreed with the previously published sequence [13] which was determined by traditional Edman degradation [15], probably due to the fact of isoforms are frequently found in plant serine protease inhibitors [26]. Further sequencing was performed after tryptic digestion of the protein, followed by MS/MS mass spectrometry and Edman sequencing of the resulting peptides.

### Enzyme Inhibition Assay

Inhibition assays with EcTI and STI were performed for a number of different enzymes. The proteases were pre-incubated for 15 min at 37°C with the inhibitors, and after that, their respective substrates were added. The assay conditions were as follows: Bovine trypsin: 7.0 nM enzyme in 0.05 M Tris/HCl, pH 8.0, 0.02% CaCl_2_, 1.0 mM BAPA; Bovine chymotrypsin: 76 nM enzyme in 0.1 M Tris/HCl, pH 8.0, 0.02% CaCl_2_; 2.0 mM Suc-Phe-pNan; Human plasma kallikrein: 67 nM enzyme in 0.05 M Tris/HCl, pH 8.0, 0.5 mM H-D-Pro-Phe-Arg-pNan; Human factor Xa: 56 nM enzyme in Tris/HCl 0.05 M, pH 8.0, 1.5 mM Boc-Ile-Glu-Gly-Arg-AMC; Human factor XIIa: 70 nM enzyme in Tris/HCl 0.05 M, pH 8.0, 1.5 mM H-D-Pro-Phe-Arg-pNan; and Human plasmin 3.5 nM enzyme in 0.1 M Tris/HCl, 0.2 M NaCl, pH 7.4, 1.0 mM H-D-Val-Leu-Lys-pNan. The Kiapp values were determined by adjusting the experimental points to the equation for tight binding, using a nonlinear regression with the Grafit program [27].

### Protein Crystallization

Initial crystallization trials of the uncomplexed EcTI were performed by using a Phoenix robot (Art Robbins Instruments, Mountain View, CA). Several hits were obtained from two different crystallization screens [JCSG Core II (Qiagen, Valencia, CA) and Crystal Screen HT (Hampton Research, Aliso Viejo, CA)]. All these hits, however, were thin needles or needle clusters, requiring extensive optimization efforts in order to improve the crystals. Eventually, diffraction quality needle-shaped crystals were yielded from condition 0.1 M MES pH 5.5, 10% MPD. Crystals appeared in several days at 20°C and grew in two weeks to a final size. For crystallization of EcTI-trypsin complex, a Phoenix robot was also used to perform the initial trials. The selected initial crystallization screens include Crystal Screen HT, JCSG II&IV, Precipitant Synergy (Emerald Biosystems, Bedford, MA), and Protein Complex Suite (Qiagen, Valencia, CA). The best hit was obtained from the Protein Complex Suite condition A7 (0.1 M ammonium sulfate, 0.1 M Tris pH 7.5, 20% PEG1500). After extensive optimization efforts, diffraction quality crystals were obtained from 0.1 M ammonium sulfate, 0.1 M Tris pH7.5, 30% PEG1500, also containing 0.1–0.4 M LiCl or 0.1–0.2 M Li_2_SO_4_. The crystals were also grown at 20°C.

### X-ray Data Collection and Processing

Diffraction data were collected at the Southeast Regional Collaborative Access Team (SER-CAT) beamline 22-ID at the Advanced Photon Source, Argonne National Laboratory. Single crystals were transferred to a cryoprotectant solution (mother liquor with extra 20% glycerol) for approximately 2 min and then were flash frozen at 100 K in a stream of liquid nitrogen. Crystals of the unbound EcTI diffracted X-rays to the resolution of 1.75 Å. Diffraction data were indexed, integrated, and scaled with the program XDS [28]. The crystal belongs to the space group *C*2 with unit cell parameters a = 160.7 Å, b = 34.9 Å, c = 65.5 Å, β = 107.7°. The asymmetric unit contains two molecules of EcTI. The estimated Matthews coefficient is 2.23 Å^3^Da^−1^, corresponding to 45% solvent content. A crystal of the complex of EcTI with trypsin diffracted X-rays to the resolution of 2.0 Å and data were also processed with the program XDS. The crystal also belongs to the space group *C*2 but with different unit cell parameters, a = 73.3 Å, b = 38.6 Å, c = 122.8 Å, β = 104.2°. Each asymmetric unit contains a single complex. The estimated Matthews coefficient is 1.74 Å^3^Da^−1^, corresponding to 29.3% solvent content. Data processing statistics for both crystal forms are shown in **Table 3**.

**Table 3:** Data collection and structure refinement.

Data collection		
	EcTI-trypsin complex	EcTI unbound
Space group	*C*2	*C*2
Molecules/a.u.	2 (1 complex)	2
Unit cell *a*, *b*, *c* (Å); β (°)	73.3, 38.6, 122.8, 104.2	160.7, 34.9, 65.5, 107.7
Resolution (Å)*	20–2.0 (2.2–2.0)	20.0–1.75 (2.0–1.75)
*R*_merge_ † (%)	8.1 (39.6)	7.8 (34.6)
No. of reflections (measured/unique)	55,037/21,100	109,420/34,536
<*I/*σ*I*>	8.63 (1.72)	10.75 (2.91)
Completeness (%)	92.1 (80.9)	97.3 (94.1)
Redundancy	2.6 (1.89)	3.2 (2.6)
**Refinement**		
Resolution (Å)	20.0–2.0	20–1.75
No. of reflections (refinement/*R*_free_)	21,100/844	34,535/1037
*R*/*R*_free_ ‡	0.196/0.258	0.183/0.237
No. atoms		
Protein	2936	2680
Ligands	5	22
Water	148	229
B-factors (Å^2^)		
Protein	36.55	28.79
Reactive loop	30.91	23.56
Ligands/water	34.51	37.78
R.m.s. deviations from ideal		
Bond lengths (Å)	0.008	0.009
Bond angles (°)	1.185	1.310
PDB accession code	4J2Y	4J2K

* The highest resolution shell is shown in parentheses.

† *R*_merge_ = ∑_h_∑_i_|*I*_i_–〈*I*〉|/∑_h_∑_i_ *I*_i_, where I_i_ is the observed intensity of the i-th measurement of reflection h, and 〈I〉 is the average intensity of that reflection obtained from multiple observations.

‡ *R* = ∑||*F_o_*|–|*F_c_*||/∑*|F_o_|,* where F_o_ and F_c_ are the observed and calculated structure factors, respectively, calculated for all data. *R*_free_ was defined in [33].

### Structure Determination and Refinement

The structure of the complex of EcTI and bovine trypsin was solved by molecular replacement with the program Phaser [29], [30]. Soybean trypsin inhibitor structure (PDB ID: 1AVW) and bovine pancreatic trypsin structure (PDB ID: 2BZA) were used as starting models. A unique solution representing a single complex in an asymmetric unit was found with data between 20 and 3 Å, with a Log-Likelihood gain of 849.7. The resulting electron density map was fully interpretable, verifying the correctness of the solution and indicating that the identity of several amino acids in the inhibitor needed to be changed. Further refinement was performed with *REFMAC5* [31] and *PHENIX* [32], using all data between 20 and 2.0 Å, after setting aside 4% of randomly selected reflections (844 total) for calculation of R_free_ [33]. Isotropic individual B-factors were refined, with the TLS parameters added in the final stages of refinement. After several further rounds of automated refinement and manual correction using COOT [34], the structural model was finally refined to an R-factor of 19.6% and R_free_ of 25.8%. The structure of free EcTI was determined by molecular replacement with *PHASER* [30] using the coordinates of EcTI part of EcTI-trypsin complex as a starting model. Refinement was carried out using a protocol similar to the one reported above for EcTI-trypsin complex. 3% randomly selected reflections (1037 total) were set aside for calculation of R_free_. The final model was refined to 1.75 Å resolution, resulting in an R-factor of 18.3% and R_free_ of 23.7% (**Table 3** **)**. The structures were compared using the *DALI* server [35] with the set of Protein Data Bank structures with less than 90% sequence identity.

### Accession Numbers

Atomic coordinates and structure factors of the refined models of free EcTI and of its complex with trypsin were deposited in the PDB with accession codes 4J2K and 4J2Y, respectively.
